# Ultrasound-Guided Nerve Hydrodissection With 5% Dextrose 4 Weeks After Steroid Injection in Treatment of Carpal Tunnel Syndrome: A Retrospective Study

**DOI:** 10.3389/fneur.2021.782319

**Published:** 2022-02-07

**Authors:** Juan-juan He, Xiao-mei Wei, Zu-lin Dou, Jiang-shan Zhang, Zhen-hai Wei, Wei-xi Zhang, Li Jiang

**Affiliations:** ^1^Department of Neurology, The First Affiliated Hospital, Sun Yat-sen University, Guangdong Provincial Key Laboratory of Diagnosis and Treatment of Major Neurological Diseases, National Key Clinical Department and Key Discipline of Neurology, Guangzhou, China; ^2^Department of Rehabilitation, The Third Affiliated Hospital, Sun Yat-sen University, Guangzhou, China; ^3^Department of Rehabilitation, The Sixth Affiliated Hospital, Sun Yat-sen University, Guangzhou, China

**Keywords:** nerve hydrodissection, ultrasound guidance, corticosteroid, dextrose, carpal tunnel syndrome

## Abstract

**Objective:**

To investigate the efficacy and safety of ultrasound-guided nerve hydrodissection (HD) with 5% dextrose (D5W) as add-on therapy after corticosteroid injection in carpal tunnel syndrome (CTS), and provide a novel strategy.

**Methods:**

In this retrospective study, patients with CTS who received ultrasound-guided nerve HD with D5W as add-on therapy after corticosteroid injection (combination group) were enrolled. Patients who received corticosteroid injection without add-on therapy (steroid group) were recruited as the control group. Ultrasound-guided nerve HD with D5W was performed 4 weeks after corticosteroid injection. Treatment effectiveness were assessed by visual analog scale (VAS) and Boston Carpal Tunnel Syndrome Questionnaire (BCTQ). The assessment was performed at baseline and 4, 8, and 12 weeks after corticosteroid injection. In addition, adverse events were recorded in this study.

**Results:**

A total of 49 patients and 62 wrists meeting the criteria were included, with 24 patients and 31 wrists in the steroid group and 25 patients and 31 wrists in the combination group. Compared with baseline data, both groups showed greater improvement in VAS, BCTQs (BCTQ severity), and BCTQf (BCTQ function) at 4, 8, and 12 weeks follow-up. VAS, BCTQs, and BCTQf scores at baseline and week 4 were comparable between steroid group and combination group. Compared with steroid group, combination group exhibited a significant reduction in VAS, BCTQs, and BCTQf at 8- and 12-week follow-up (*P* ≤ 0.01). No adverse event occurred in any group.

**Conclusions:**

Our results showed that ultrasound-guided nerve HD with D5W as add-on therapy after corticosteroid injection was efficacious and safe in CTS, and combination therapy is more beneficial than corticosteroid monotherapy in the improvement of symptoms and function at 8- and 12-week follow-up.

## Introduction

Carpal tunnel syndrome (CTS) is the most common peripheral entrapment neuropathy, with an annual incidence rate of 99 in 100,000 people (1). It is well-known that 1 in 10 people develop CTS at times, most of whom are older women (2). CTS results in gradual ischemia and damage of the median nerve (MN). Therefore, pain, paresthesia, numbness, tingling, and hand weakness are the most common symptoms.

CTS can be treated with various methods, such as perineural injection with corticosteroid, especially for the patients who failed conventional non-invasive approaches (3). However, some researchers reported that the improvement was most obvious at 1 month after injection with corticosteroid, then the therapeutic effect declined over time, or even reversed through the 3rd to the 6th month after injection (4, 5). In our clinical practice, we observed that patients still had a certain degree of discomfort, such as numbness and pain, at 4 weeks after perineural injection with corticosteroid. Some patients requested an add-on therapy to relieve symptoms thoroughly. Therefore, we gave an add-on HD with 5% dextrose (D5W) to patients who were willing to receive it. Wu found that perineural injection of D5W is safe and effective for CTS both in the short term and in the long term (5).

As to the perineural injection technique, ultrasound-guided nerve hydrodissection (HD) (6) is a novel method in recent years, and multiple randomized controlled trials have demonstrated its effectiveness and safety (7–9). Nerve HD involves using an anesthetic or solution to separate the nerve from the surrounding tissue, fascia, or adjacent structures (10), which is believed to constrict or irritate the nerve either during movement or at rest. Several kinds of injectate can be selected for ultrasound-guided nerve HD. For example, corticosteroid and dextrose are commonly used (7–9).

As mentioned above, perineural injection with corticosteroid is effective for the treatment of CTS, but patients still had some clinical symptoms in varying degrees of severity at 4 weeks after injection. So we gave an add-on HD with D5W to some patients in our clinic practice. We want to explore the efficacy and safety of HD with D5W as add-on therapy after corticosteroid injection in CTS, and provide a novel strategy for the treatment of CTS.

## Materials and Methods

### Design

This study was a retrospective study performed at a single center. We retrospectively searched the medical records from a nerve HD database at Rehabilitation Department of the Third Affiliated Hospital of Sun Yat-sen University, from January 2017 to September 2021. According to the treatment protocols, patients were divided into two groups, steroid group and combination group. Patients in the combination group received corticosteroid injection and ultrasound-guided nerve HD with D5W as add-on therapy 4 weeks later. Patients in the steroid group only received corticosteroid injection without add-on therapy. Inclusion criteria: (1) patients diagnosed with CTS and had insufficient responses to conventional therapy (rest, physical therapy, splint, and oral analgesic), then received one of the above treatment protocols; (2) between 30 and 80 years old; (3) with complete baseline or follow-up data; (4) no additional treatment for CTS was delivered during the follow-up period; (5) without other diseases that affect MN, such as polyneuropathy or diabetic peripheral neuropathy. The diagnosis of CTS includes associated signs and symptoms (5, 11) such as nocturnal, postural, or usage associated paresthesia of the affected hand, and positive Tinel or Phalen sign, and confirmed by electrophysiological test no matter where conducted. Insufficient response was defined as after conventional therapy, numbness intensity was ≥3, measured by visual analog scale (VAS). Exclusion criteria: (1) therapy carried out not according to any of the above protocols; (2) a lack of baseline or follow-up data; (3) over 80 years old or <30 years old; (4) received corticosteroid injection for CTS 3 months before initial HD; (5) received additional treatment for CTS during the follow-up period; (6) a history of other neurologic diseases that would affect MN.

All outcomes were measured at baseline and the end of 4-, 8-, and 12-week from baseline. A single investigator obtained clinical history and performed physical examinations and CTS assessment.

### Interventions

The patients were administered 1 or 2 sessions of perineural injection under the high-resolution musculoskeletal ultrasound device (S-Nerve Ultrasound System, P07576, Sonosite, Bothell, WA, USA; transducer, HFL38x/13-6 MHz, Sonosite, Bothell, WA, USA). Patients in both groups received 1 session of HD with 0.5 ml compound betamethasone (5 mg betamethasone dipropionate phosphate/2 mg betamethasone sodium phosphate in 1 ml, Schering-Plough Labo N.V., Belgium) mixed with 4.5 ml 0.9% normal saline. Patients in the combination group received the second session of HD with 5 ml D5W (dextrose injection 5%, Shijiazhuang Siyao Co. Ltd, Shijiazhuang city, Hebei province, China) 4 weeks after the HD with corticosteroid. The second session of HD with D5W was performed 1 h after assessment of VAS and BCTQ at 4-week follow-up.

**Figure 1:**
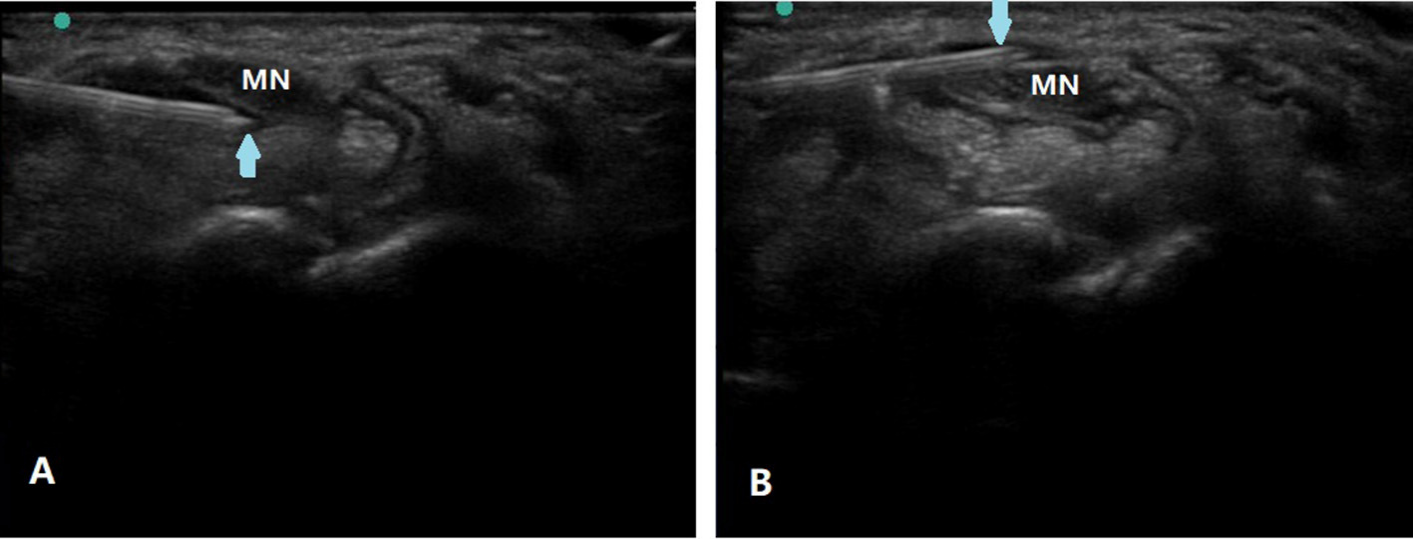
Ultrasound-guided nerve HD of the MN in CTS. Using in-plane ulnar approach, partial injectate was injected to hydrodissect the inferior MN away from the flexor tendons **(A)** and the residual injectate was then injected to hydrodissect the MN from the flexor retinaculum **(B)**. Arrow, puncture needle; MN, median nerve.

The MN was identified at the proximal inlet of the carpal tunnel. Under in-plane ulnar approach (12), 2 ml injectate was injected to hydrodissect the inferior MN away from the flexor tendons and the residual 3 ml injectate was then injected to hydrodissect the MN from the flexor retinaculum (Figure 1). After injection with corticosteroid or D5W, the operator scanned through the whole carpal tunnel to confirm the injectate around MN (5). Every patient was observed for half an hour after injection for any complications, such as swelling, pain, bleeding, increased paresthesia, or numbness before discharge.

### Outcome Measurements

The outcomes were measured by the VAS and the Boston Carpal Tunnel Syndrome Questionnaire (BCTS), which are the most commonly used measurements for CTS. VAS was used to indicate the intensity of average level of numbness within the past 1 week. We found that numbness was the most common chief complaint of Chinese patients with CTS, so VAS was used to assess the severity of numbness. VAS ranges from 0 to 10, with 0 as no numbness and 10 as the most severe numbness ever experienced. BCTQ includes 2 subscales, assessment of symptoms (BCTQs) and functions (BCTQf), respectively. There are 11 questions on symptom severity and 8 questions on functional status, with scores ranging from 0 to 5 for each question. A score of 0 means mildest symptoms or no difficulty in activity, and a score of 5 means very severe symptoms and cannot perform the activity at all (5).

### Sample Size

G^*^power 3.1.9.7 (University of California, Los Angeles) was used to calculate sample size in this repeated-measures analysis of variance for comparison of two groups. For an effect size ranging from 0.5 to 0.25, data for at least 14–48 wrists were required to achieve sufficient power [(1-β) = 0.95 and α = 0.05] (7).

### Data Analysis

Statistical analysis of the data was performed for all collected data using SPSS statistics version 26 (IBM, Armonk, NY). Independent *t*-test and chi-squared test or Fisher exact test were used to analyze continuous and categorical demographic data, respectively. Paired Student's *t*-test was used to assess the changes in VAS and BCTQ scores between baseline and different follow-up time points. Repeated-measures analysis of variance (ANOVA) with subsequent *post-hoc* Bonferroni test was used to compare within-group changes in VAS and BCTQ scores over time (from weeks 4 to 12) and group (combination group vs. steroid group) × time (weeks 4, 8, and 12) interaction effects. The statistical tests were 2-tailed, and a *P*-value < 0.05 was considered statistically significant. Bonferroni-corrected values of *P* < 0.0167 (0.05/3 time points) for the inter-group comparisons and *P* < 0.0167 (0.05/3 comparisons) for the intra-group comparisons were considered statistically significant to avoid inflated type I errors.

## Results

### The Baseline Data of Enrolled Cases

A total of 49 patients, with 62 wrists met the criteria for inclusion. The baseline demographics and clinical characteristics are summarized in Table 1. No significant differences were found at baseline between the two groups (Tables 1, 2).

**Table 1:** Baseline demographic and clinical characteristics of study.

**Characteristic**	**Steroid group, *n* = 31 (patient, *n* = 24)**	**Combination group, *n* = 31 (patient, *n* = 25)**	***P^**a**^***
**Gender**, ***n*** **(%)**			
Female	25 (80.6)	25 (80.6)	1.0
Male	6 (19.4)	6 (19.4)	
Age, mean ± SD	54.8 ± 9.1	55.6 ± 13.3	0.781
BMI, mean ± SD	25.7 ± 2.0	25.6 ± 1.1	0.772
**Lesion site**, ***n*** **(%)**			
Left	11 (35.5)	10 (32.3)	0.788
Right	20 (64.5)	21 (67.7)	
**Unilateral or bilateral**, ***n*** **(%)**			
Unilateral	24 (77.4)	25 (80.6)	0.755
Bilateral	7 (22.6)	6 (19.4)	
**Duration**, ***n*** **(%)**			
≤ 1 year	19	16	0.842
>1 year, ≤ 10 years	10	13	
>10 years	2	2	

a *Independent t-test, chi-squared test, or Fisher exact test*.

**Table 2:** Comparison of VAS and BCTQ scores at baseline and week 4 between groups.

	**Baseline**			**Week 4**		
	**VAS, mean ±SD**	**BCTQs, mean ±SD**	**BCTQf, mean ±SD**	**VAS, mean ±SD**	**BCTQs, mean ±SD**	**BCTQf, mean ±SD**
Steroid group	6.1 ± 1.6	28.7 ± 5.2	19.3 ± 3.5	4.2 ± 1.4	21.1 ± 3.9	15.0 ± 2.4
Combination group	6.3 ± 1.6	29.3 ± 4.9	18.6 ± 4.1	4.1 ± 1.5	20.8 ± 4.1	14.6 ± 2.5
*P*	0.691	0.670	0.446	0.794	0.729	0.564

*VAS, visual analog scale; BCTQ, Boston Carpal Tunnel Syndrome Questionnaire (f, function; s, severity)*.

### The Effect of Different Interventions on Patients

Compared with baseline data, both groups showed greater improvement in VAS, BCTQs, and BCTQf scores at different follow-up points (*P* < 0.01) (Table 3). In addition, VAS, BCTQs, and BCTQf scores at weeks 4 were comparable between steroid group and combination group (*P* > 0.05) (Table 2).

**Table 3:** The outcome variables (VAS and BCTQ) before and after treatment in both groups.

	**Steroid group, *n* = 31, mean ±SE**	***p***	**Combined group, *n* = 31, mean ±SE**	***p***
VAS baseline	6.1 ± 1.6		6.3 ± 1.6	
Week 4	4.2 ± 1.4	<0.001	4.1 ± 1.5	<0.001
Week 8	3.8 ± 1.3	<0.001	2.8 ± 1.3	<0.001
Week 12	3.6 ± 1.4	<0.001	2.3 ± 1.4	<0.001
BCTQs baseline	28.7 ± 5.2		29.3 ± 4.9	
Week 4	21.1 ± 3.9	<0.001	20.8 ± 4.1	<0.001
Week 8	19.5 ± 4.0	<0.001	16.6 ± 3.2	<0.001
Week 12	19.0 ± 3.5	<0.001	14.8 ± 2.8	<0.001
BCTQf baseline	19.3 ± 3.5		18.6 ± 4.1	
Week 4	15.0 ± 2.4	<0.001	14.6 ± 2.5	<0.001
Week 8	13.4 ± 2.3	<0.001	11.9 ± 2.3	<0.001
Week 12	12.6 ± 2.3	<0.001	11.4 ± 2.2	<0.001

*VAS, visual analog scale; BCTQ, Boston Carpal Tunnel Syndrome Questionnaire (f, function; s, severity)*.

**Figure 2:**
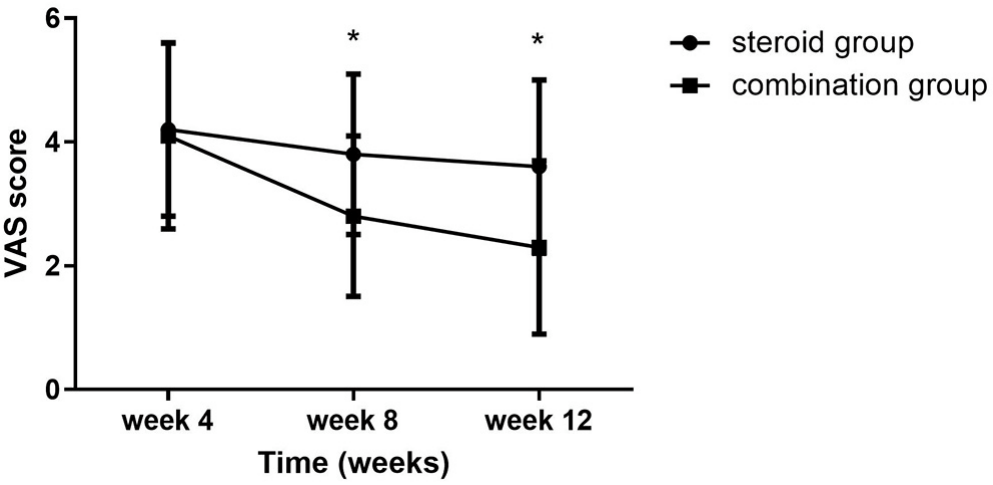
VAS at 4-, 8-, and 12-week follow-up between the steroid and combination groups (mean ± SD). The differences were significant at weeks 8 and 12 (*P < 0.01, ANOVA with subsequent *post-hoc* Bonferroni test was used). VAS, visual analog scale.

**Figure 3:**
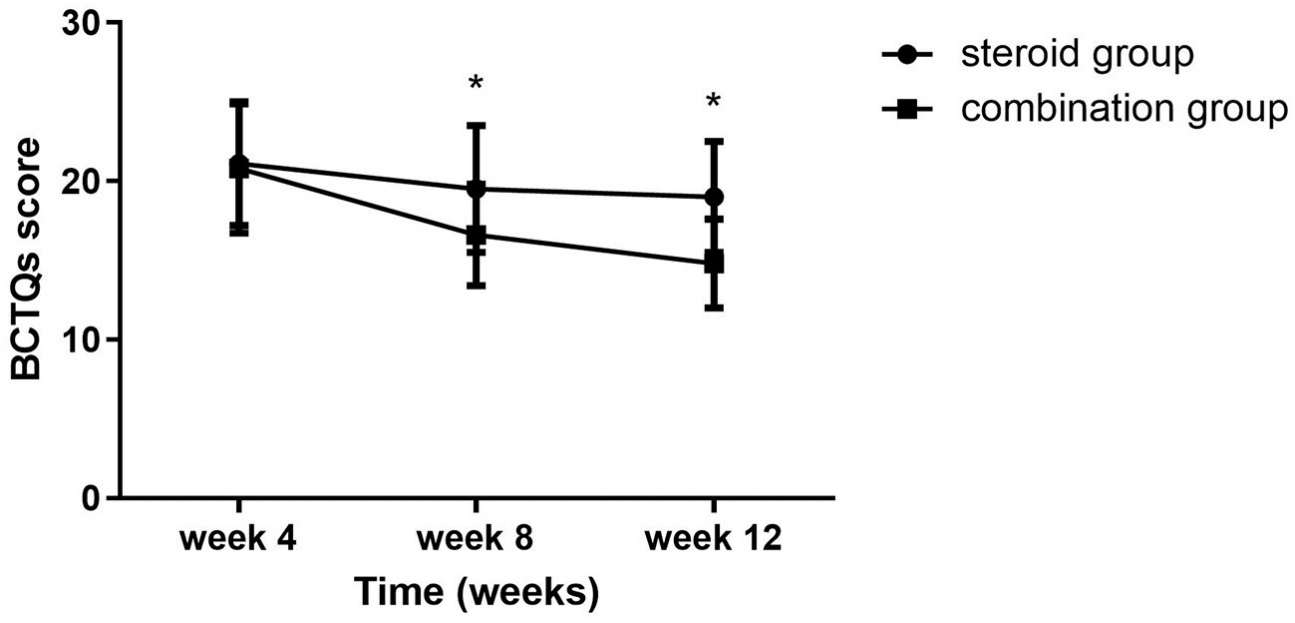
BCTQs at 4-, 8-, and 12-week follow-up between the steroid and combination groups (mean ± SD). The differences were significant at weeks 8 and 12 (*P < 0.01, ANOVA with subsequent *post-hoc* Bonferroni test was used). BCTQ, Boston Carpal Tunnel Syndrome Questionnaire; s, severity.

**Figure 4:**
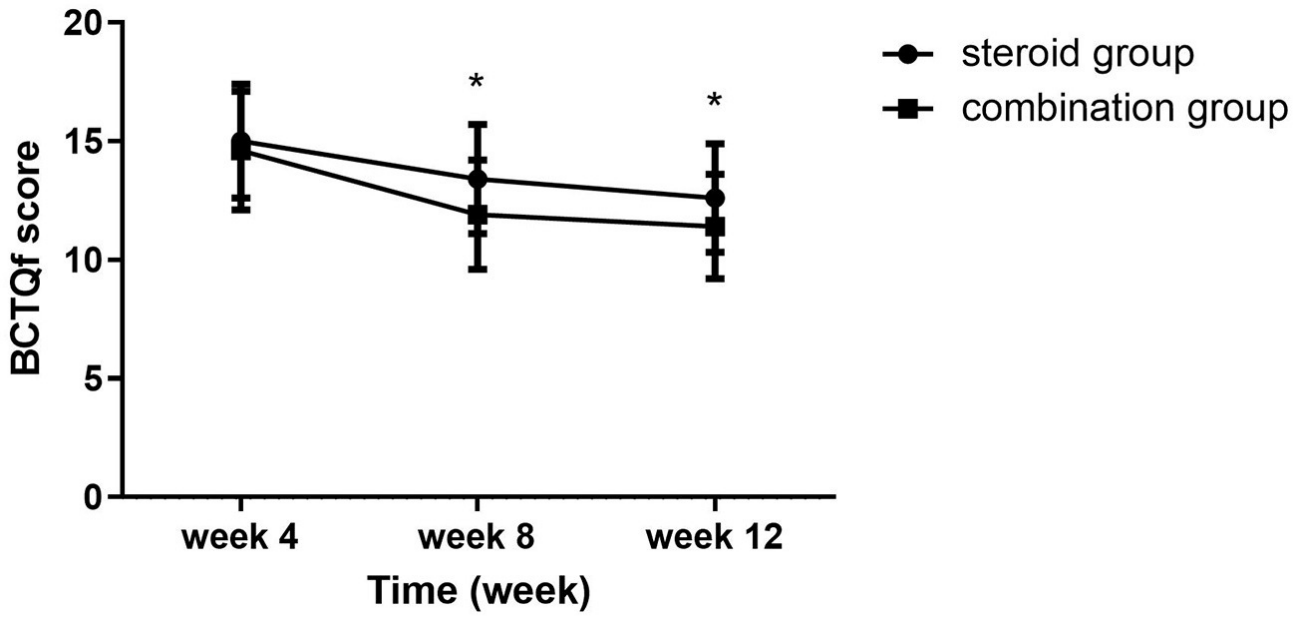
BCTQf at 4-, 8-, and 12-week follow-up between the steroid and combination groups (mean ± SD). The differences were significant at weeks 8 and 12 (*P ≤ 0.01, ANOVA with subsequent *post-hoc* Bonferroni test was used). BCTQ, Boston Carpal Tunnel Syndrome Questionnaire; f, function.

Significant group (combination group versus steroid group) × time (weeks 4, 8, and 12) interaction effects were observed in VAS (*F* = 6.467, *P* = 0.003), BCTQs (*F* = 15.878, *P* = 0.000), and BCTQf (*F* = 3.543, *P* = 0.035). When compared with steroid group, VAS, BCTQs, and BCTQf scores of combination groups declined significantly at weeks 8 and 12 (*P* < 0.01) (Figures 2–4).

Intra-group comparison of VAS scores did not display any significant changes in steroid group from weeks 4 to 12. However, VAS scores continued to decline from week 4 to week 8 in the combination group (*P* < 0.01). BCTQs scores decreased from weeks 4 to 8 in the steroid group (*P* < 0.01) but remained essentially unchanged from weeks 8 to 12 (*P* > 0.05). A noteworthy finding was that BCTQs scores decreased from weeks 4 to 8 and continued to decline to week 12 in the combination group (*P* < 0.01). Similar to the finding for BCTQs, BCTQf scores decreased from weeks 4 to 8 in the steroid group (*P* < 0.01), then remained unchanged from weeks 8 to 12 (*P* > 0.05). Although BCTQf scores decreased from weeks 4 to 8 (*P* < 0.01), and continued to decline to week 12 in combination group, the difference did not reach statistic difference (*P* > 0.05).

There were no side effects or complications observed in this study.

## Discussion

In this study, we explored the efficacy and safety of HD with D5W as add-on therapy after corticosteroid injection in the treatment of CTS, and compared the therapeutic effect with corticosteroid monotherapy. After interventions, significant improvements in symptoms and function were observed at all the follow-up time points in both groups. In addition, the combination group exhibited significant reduction in the severity of symptoms and dysfunction, compared to the steroid group at 8- and 12-week follow-up.

Several clinical studies have confirmed the efficacy and safety of perineural injection with corticosteroid in CTS (4, 5, 11). In the current study, both groups showed considerable improvements in symptoms and functions at 4 weeks after corticosteroid injection. In a review conducted by Marshall et al. the authors found that local corticosteroid injection for CTS provides greater clinical improvement in symptoms 1 month after injection compared to placebo. However, symptom relief beyond 1 month compared to placebo has not been demonstrated (13). Lee reported that BCTQ showed significant improvement at 4 weeks after ultrasound-guided local corticosteroid injection in the CTS, and the improvement was still observed at 12 weeks (12). Our findings were compatible with the above studies at 4 weeks, but no further improvement in VAS scores was observed from 4 to 12 weeks in the steroid group, indicating that an add-on therapy may be a strategy for enhancing the therapeutic efficacy of CTS.

Marshall revealed that two local corticosteroid injections do not provide significant added clinical benefit compared to one injection for CTS; besides, repeated injection of steroid results in possible nerve damage (14). So in this study, we combined corticosteroid with D5W in CTS. Wu demonstrated that perineural injection of D5W is more beneficial than that of corticosteroid in patients with mild-to-moderate CTS at 4–6 months postinjection, but there was no significant difference in VAS and BCTQs between two groups within initial 3 months (5). Our results showed that the improvement of symptoms in the combination group was more significant compared to steroid group at 8 and 12 weeks after initial HD, indicating that combination therapy provided better efficacy than corticosteroid monotherapy in a short time and implying that symptoms may continue to improve gradually over time in the combination therapy. We performed HD with D5W 4 weeks after corticosteroid injection, because it is reported that active metabolite of betamethasone dipropionate has a half-life of ~3 days (15) and takes more than 10 days for excretion. So HD with D5W performed 4 weeks later would not interact with corticosteroid.

As mentioned above, the role of corticosteroid is well-established. However, the definite mechanism underlying the effectiveness of D5W is still not clear. It is hypothesized that dextrose could decrease neurogenic inflammation by inhibiting transient receptor potential vanilloid receptor-1 (TRPV1) and different neurotransmitters (16, 17), such as calcitonin gene-related peptide and substance P. However, further *in vivo* and *in vitro* studies are needed to explore the exact mechanisms of D5W.

In the studies conducted by Wu, no associated complications with HD were reported, no matter HD with corticosteroid or D5W (5, 7). Also no adverse effect was observed throughout 12 weeks follow-up period in this study. This suggests that HD with D5W as add-on therapy in CTS is safe and feasible. Maybe the combination therapy can be considered by clinicians in the treatment of CTS.

However, difference in BCTQf between combination group and steroid group was very modest despite statistically significance. A possible reason is that function restoration depends on many factors, and HD with corticosteroid had already exerted considerable effect at 4 weeks, so in a short term, from 4 to 12 weeks, the differences in functional improvement were not significant. Thus, a prospective study with longer observation time is still needed to warrant the result of this study in the future.

In the current study, we performed ultrasound-guided HD with corticosteroid or D5W using an in-plane ulnar approach. Reportedly, ultrasound-guided local corticosteroid injection using an in-plane ulnar approach in the CTS can be more effective than other approaches (12). There's no consensus on the injectate volume and frequency of HD in CTS currently. In this study, the volume of 5 ml was selected for HD. In most of the studies, injection volume on HD in CTS is 5 ml (5, 7). Some researchers believed that a much larger volume of injectate needs to be used to completely release the nerve from surrounding tissue to achieve an oval appearance (10). Wu observed greater effectiveness with repetitive injection in their clinical practice (5). Lam also recommended addition of second injection after initial HD (18). As to the frequency of HD, maybe once every 2–6 weeks (18, 19). So in this study, we chose 4 weeks after initial HD for a second session of HD, in order to enhance the effectiveness.

## Limitations

This study has a few limitations. First, this is a retrospective study and the sample size is relatively small. Second, nerve electrophysiology was not used for assessment of therapeutic effect. It takes a long time to make an appointment for electrophysiological tests in our hospital, so some patients were examined in other hospitals or examined not on time. As a result, the data of nerve electrophysiology are not collected and analyzed in this study. Third, the follow-up duration is short. Because the patients included were outpatients, if symptoms improved significantly, they would not insist on follow-up visits. Fourth, in the present study, injection was performed twice in the combination group, but only performed once in the corticosteroid group. Placebo effect may exist in the combination group. In future studies, we will design a second injection for the corticosteroid group, in order to exclude a placebo effect.

## Conclusion

In conclusion, this study showed that ultrasound-guided nerve HD with D5W as add-on therapy after corticosteroid injection was efficacious and safe in CTS, and combination therapy is more beneficial than corticosteroid monotherapy at 8- and 12-week follow-up in the improvement of symptoms and functional status. Maybe combination therapy provides a novel strategy for the treatment of CTS.

## Data Availability Statement

The raw data supporting the conclusions of this article will be made available by the authors, without undue reservation.

## Ethics Statement

The studies involving human participants were reviewed and approved by the Ethics Committee of the Third Affiliated Hospital of Sun Yat-sen University. The ethics committee waived the requirement of written informed consent for participation.

## Author Contributions

LJ and W-xZ designed the investigation. J-jH and X-mW performed the investigation and wrote the manuscript. LJ and Z-lD revised the manuscript. J-sZ and Z-hW contributed to data collection and analyses. All authors have read and approved the manuscript. All authors contributed to the article and approved the submitted version.

## Funding

The study was supported by the grants from: Guangdong Provincial Key Laboratory for Diagnosis and Treatment of Major Neurological Diseases (2017B030314103), the Southern China International Cooperation Base for Early Intervention and Functional Rehabilitation of Neurological Diseases (2015B050501003), Guangdong Provincial Engineering Center for Major Neurological Disease Treatment, Guangdong Provincial Translational Medicine Innovation Platform for Diagnosis and Treatment of Major Neurolgoical Disease, Guangdong Province Key Research and Development Plan (2018B030339001), and a crosswise project of First Affiliated Hospital, Sun Yat-sen University (2020027).

## Conflict of Interest

The authors declare that the research was conducted in the absence of any commercial or financial relationships that could be construed as a potential conflict of interest.

## Publisher's Note

All claims expressed in this article are solely those of the authors and do not necessarily represent those of their affiliated organizations, or those of the publisher, the editors and the reviewers. Any product that may be evaluated in this article, or claim that may be made by its manufacturer, is not guaranteed or endorsed by the publisher.
